# Sensitive and less invasive confirmatory diagnosis of visceral leishmaniasis in Sudan using loop-mediated isothermal amplification (LAMP)

**DOI:** 10.1371/journal.pntd.0006264

**Published:** 2018-02-14

**Authors:** Maowia Mukhtar, Sababil S. Ali, Salah A. Boshara, Audrey Albertini, Séverine Monnerat, Paul Bessell, Yasuyoshi Mori, Yutaka Kubota, Joseph M. Ndung’u, Israel Cruz

**Affiliations:** 1 Institute of Endemic Diseases, University of Khartoum, Khartoum, Sudan; 2 Foundation for Innovative New Diagnostics—FIND, Geneva, Switzerland; 3 Eiken Chemical Co., Ltd., Tokyo, Japan; Institut de Recherche pour le Développement, FRANCE

## Abstract

**Background:**

Confirmatory diagnosis of visceral leishmaniasis (VL), as well as diagnosis of relapses and test of cure, usually requires examination by microscopy of samples collected by invasive means, such as splenic, bone marrow or lymph node aspirates. This causes discomfort to patients, with risks of bleeding and iatrogenic infections, and requires technical expertise. Molecular tests have great potential for diagnosis of VL using peripheral blood, but require well-equipped facilities and trained personnel. More user-friendly, and field-amenable options are therefore needed. One method that could meet these requirements is loop-mediated isothermal amplification (LAMP) using the Loopamp *Leishmania* Detection Kit, which comes as dried down reagents that can be stored at room temperature, and allows simple visualization of results.

**Methodology/Principal findings:**

The Loopamp *Leishmania* Detection Kit (Eiken Chemical Co., Japan), was evaluated in the diagnosis of VL in Sudan. A total of 198 VL suspects were tested by microscopy of lymph node aspirates (the reference test), direct agglutination test-DAT (in house production) and rK28 antigen-based rapid diagnostic test (OnSite *Leishmania* rK39-Plus, CTK Biotech, USA). LAMP was performed on peripheral blood (whole blood and buffy coat) previously processed by: i) a direct boil and spin method, and ii) the QIAamp DNA Mini Kit (QIAgen). Ninety seven of the VL suspects were confirmed as cases by microscopy of lymph node aspirates. The sensitivity and specificity for each of the tests were: rK28 RDT 98.81% and 100%; DAT 88.10% and 78.22%; LAMP-boil and spin 97.65% and 99.01%; LAMP-QIAgen 100% and 99.01%.

**Conclusions/Significance:**

Due to its simplicity and high sensitivity, rK28 RDT can be used first in the diagnostic algorithm for primary VL diagnosis, the excellent performance of LAMP using peripheral blood indicates that it can be also included in the algorithm for diagnosis of VL as a simple test when parasitological confirmatory diagnosis is required in settings that are lower than the reference laboratory, avoiding the need for invasive lymph node aspiration.

## Author summary

Tissue aspiration, either from spleen, bone marrow or lymph node, remains the Gold Standard for parasitological confirmation in patients suspected of visceral leishmaniasis (VL), and is often used for detection of relapses, and as a test of cure. The procedure is invasive, with risks of severe complications, requires skilled personnel to perform, and appropriate facilities to manage severe adverse events, if they occur. These drawbacks can be solved by using sensitive diagnostic test based on peripheral blood. Nucleic acid amplification tests (NAAT) are sensitive for the detection of *Leishmania* parasites in blood; however, in VL-endemic settings, most NAAT are restricted to well-equipped laboratories. A robust NAAT, Loopamp *Leishmania* Detection Kit has recently been developed in a collaboration between FIND, Eiken Chemical Co. Ltd., Japan and other partners. We have evaluated this kit in Sudan and obtained a sensitivity of 97.6% and specificity of 99.1%, using DNA obtained from peripheral blood through a simple boil and spin method. Its simplicity and excellent diagnostic performance make this kit ideal for parasitological confirmation of VL in less equipped laboratories.

## Introduction

Visceral leishmaniasis (VL), also known as kala-azar, is a vector-borne disease caused by parasitic protozoa of the *Leishmania donovani* complex, which are transmitted by female sandflies. VL affects mainly children and young adults, and can be fatal if left untreated. It is characterized by fever, weight loss, wasting, and splenomegaly; lymphadenopathy is especially frequent in VL patients in Sudan, where it can be the only clinical manifestation accompanying fever [1].

Sudan ranks third among the VL high-burden countries after India and South Sudan, which together with Bangladesh, Brazil and Ethiopia account for 90% of VL cases worldwide, according to recent WHO estimates [2]. Access to accurate diagnosis is one of the main challenges for VL control in Sudan, where out of the 3,520 VL cases reported in 2014, only 62% of them had a confirmatory diagnosis in the laboratory [2–4].

Usually, the first approach for laboratory diagnosis of cases suspected of VL in Sudan is serology, using either the direct agglutination test (DAT) or rK39-based rapid diagnostic tests (RDT). The second approach is lymph node aspirate microscopy. Lymphadenopathy is an important sign in VL patients in Sudan, and enlarged nodes are usually found during clinical examination. Despite its limited sensitivity (52–65%), examination of lymph node aspirates is preferred over bone marrow or spleen aspirates because it is easier and safer, and can be performed by paramedical staff. Examination of bone marrow or splenic aspirates improves sensitivity (75–95%), but this requires experienced personnel, and should be performed in hospitals where blood transfusion is available, which might be needed in case of an adverse event. The accuracy of microscopic examination is also influenced by the ability of the laboratory technician and the quality of the reagents used. Bone marrow and splenic aspiration, however, are rarely performed in Sudan due to technical and logistical difficulties, and are limited to reference laboratories [3,5,6].

Parasite confirmation by tissue aspirate microscopy is also used for test-of-cure, since serology is useless for this purpose as anti*-Leishmania* antibodies remain detectable up to several years after cure [1,7]. Thus, this is another scenario where approaches to confirm *Leishmania* infection that are less invasive are required. In recent years molecular tests such as the polymerase chain reaction (PCR) and quantitative real-time PCR, when performed on peripheral blood, have shown high sensitivity and specificity in the diagnosis of VL and treatment monitoring [8–11]. Unfortunately, these require well-equipped facilities and trained personnel, and since in most cases in-house protocols are used, there is an important lack of standardisation, limiting their use to reference laboratories. To make molecular diagnosis available in low resource settings, more user-friendly, design-locked and field-amenable options are therefore needed. One method that could meet such criteria is loop-mediated isothermal amplification (LAMP) of DNA: it is performed at constant temperature (60–65°C) for target DNA amplification rather than thermocycling; is highly specific, based on a set of four primers recognizing six distinct regions on the target; it has high amplification efficiency and enables amplification within a short time; results can also be visualized using simple detection methods. Therefore, LAMP has emerged as a powerful tool for point-of-care diagnosis [12,13]. A LAMP assay, Loopamp *Leishmania* Detection Kit (Eiken Chemical Co., Japan), based on dried down reagents immobilised in the reaction tube that can be stored at room temperature, was developed in a collaboration between Eiken Chemical Co., FIND and partners. In the present study, we report on the evaluation of the performance of Loopamp *Leishmania* Detection Kit in the diagnosis of VL using peripheral blood in Sudan.

## Methods

### Study sites

The study was carried out between December 2014 and February 2016 at two sites in Sudan: Bazoura Rural Hospital (BRH) in Gedaref State, and the Institute of Endemic Diseases (IEND) in Khartoum.

### Study participants

Patients presenting themselves at BRH or referred to IEND, presenting clinical signs and symptoms suggestive of VL: fever of more than two weeks plus either weight loss, splenomegaly or lymphadenopathy, were considered VL suspects. Once malaria was ruled out by microscopy of finger prick blood films the patients were prospectively and consecutively enrolled in the study if they fulfilled additional inclusion criteria: i) provision of informed consent and ii) judged possible to obtain peripheral blood and lymph node aspirates. Patients with previous history of VL or being under treatment for VL, pregnant women, patients in extremis or having severe concomitant illness, and those unable to provide informed consent were excluded from the study.

The sample size was designed around estimating the sensitivity of Loopamp *Leishmania* Detection Kit with a binomial error margin of ±8%. Assuming a prevalence of 45% and a LAMP sensitivity of 85%, we estimated that 192 VL suspects would be recruited, thus we aimed to recruit 200 VL suspects.

### Ethical considerations

The study was carried out in conformity with the Helsinki Declaration, and ethical approval was obtained from the Research Ethics Committee of the Institute of Endemic Diseases, University of Khartoum (Reference N°: 1/2014). Participants were informed about the objectives and procedures of the study, and benefits and risks were also explained. Written informed consent was obtained from all participants /parents or guardians in the presence of independent witnesses before collecting samples. Confidentiality was assured by assigning a study code to each participant, and patient information was kept controlled at each of the study sites following IEND requirements.

The full study protocol can be accessed by request to the authors.

### Sample collection

Five ml peripheral blood was collected from each patient, both in Serum Separator and Heparin Vacutainer tubes (Becton Dickinson). Buffy coat was obtained from 3 ml heparin-treated peripheral blood by centrifugation at 8,000 X g; the buffy coat was transferred to an Eppendorf tube and re-suspended in residual plasma to allow for a 200–250 μl volume buffy coat suspension. Whole blood, buffy coat and serum aliquots were kept at -20°C until analysis.

Lymph node aspirates were taken from enlarged lymph nodes, and a Giemsa stained smear was prepared for microscopy.

When collected at BRH, whole blood and serum samples were periodically transferred to IEND during the study, maintaining a cold chain. After examination, smears of lymph node aspirates were also transferred to IEND, where they were examined again.

### Microscopy

Giemsa stained smears were examined using Primo Star microscopes (Carl Zeiss Microscopy GMBH, Germany). *Leishmania* amastigotes were confirmed under 1000x magnification. Lymph node aspirate microscopy (LNA-M) was considered as the reference test of the study.

### rK28 rapid diagnostic test (RDT)

Serum samples (50 μl) were tested with the rK28-based RDT OnSite *Leishmania* rK39-Plus (CTK Biotech, Inc., USA) following the manufacturer’s instructions. This test is based on the rK28 antigen, a chimeric protein composed by the first two 39 amino acid repeats of the Sudanese kinesin homologue LdK39 flanked by the repeat sequences of HASPB1 (or K26) and the whole open reading frame of HASPB2 (or K9) [14]. Results were recorded after 15 minutes, and whenever an invalid result was obtained, the test was repeated immediately.

### Direct agglutination test (DAT)

Serum samples were tested by an in house DAT produced at IEND following the procedure described by Harith *et al*. [15]. Samples with a titre ≥1:3,200 were considered as positive. Titres of 1:800 and 1:1600 were considered as borderline.

### DNA extraction

Peripheral blood samples were processed following two different methods prior to LAMP analysis:

*Boil & Spin*: 95 μl heparin-treated whole blood or 95 μl buffy coat were mixed with 5 μl 10% SDS by inversion 10 times in a 1.5 ml Eppendorf tube, allowed to stand for 10 min at room temperature (RT) and mixed again. After adding 400 μl PCR grade H_2_O the mixture was incubated in a heating block at 90°C for 10 min. The mixture was spun down for 3 min at maximum speed in a bench top centrifuge (13,000 rpm), the supernatant was recovered and processed immediately or stored at -20°C until analysis by LAMP.

*QIAamp DNA Mini kit (QIAGEN)*: 200 μl heparin-treated whole blood or 100 μl buffy coat were processed following the instructions provided in the kit. The DNA was eluted in 200 μl PCR grade water and processed immediately or stored at -20°C until analysis by LAMP.

### Loopamp *Leishmania* Detection Kit (LAMP)

**Fig 1:**
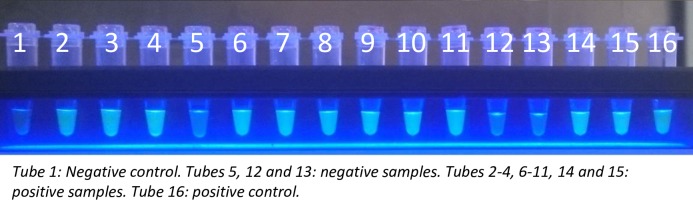
Identification of positive and negative LAMP results using blue LED light illumination with a visualization unit that is integral to the Loopamp LF-160 incubator.

The Loopamp *Leishmania* Detection Kit uses primers targeting two different regions of the *Leishmania* genome: the *18S rRNA* gene and the kDNA minicircles, and is specific to the *Leishmania* genus. *Bst* DNA polymerase is used for amplification, and the dried reagents include calcein to allow for the visual judgement of the amplified products without opening the reaction tubes. The kit includes negative and positive controls; the positive control is an artificial construct based on DNA sequences from *L*. *donovani* isolates from India and Sudan (GenBank Accession Numbers Y11401 and X07773). Three μl of the DNA obtained by the two DNA extraction methods described above were used for the LAMP reaction. This was run for 40 min at 65°C in the Loopamp LF-160 incubator (Eiken Chemical Co., Japan), the results were visualized under blue LED light illumination, using a visualization unit that is integral to the incubator. Fig 1 shows an example of positive and negative samples visualized under blue LED light illumination. LAMP reactions were performed after the other diagnostic tests, by a technician who was blinded from the results of these tests.

### Statistical analysis

Participants who were positive by LNA-M were considered VL cases, while all other subjects were considered non-VL cases. Epidat 3.0 was used to calculate sensitivity, specificity, positive and negative predictive values, and concordance between tests (Cohen’s kappa coefficient) [16]. The false discovery rate (FDR) was calculated as 1—the positive predictive value (PPV) and the false omission rate (FOR) as 1—the negative predictive value (NPV), considering the calculated sensitivity and specificity. Where sensitivity or specificity was calculated to be 100%, an arbitrary value of 99.9% was used for illustrative purposes. The FDR and FOR were plotted against prevalences ranging from 0–100% to illustrate their change over the range of potential disease contexts. FDR and FOR were chosen as they are more directly applicable to field studies than PPV and NPV. This approach assumes that sensitivity and specificity do not change with prevalence. McNemar’s test was applied to compare data from LAMP with different sample types (buffy coat vs. whole blood) and sample preparation methods (QIAgen vs. Boil & Spin).

A STARD Checklist is provided as S1 Table.

## Results

**Fig 2:**
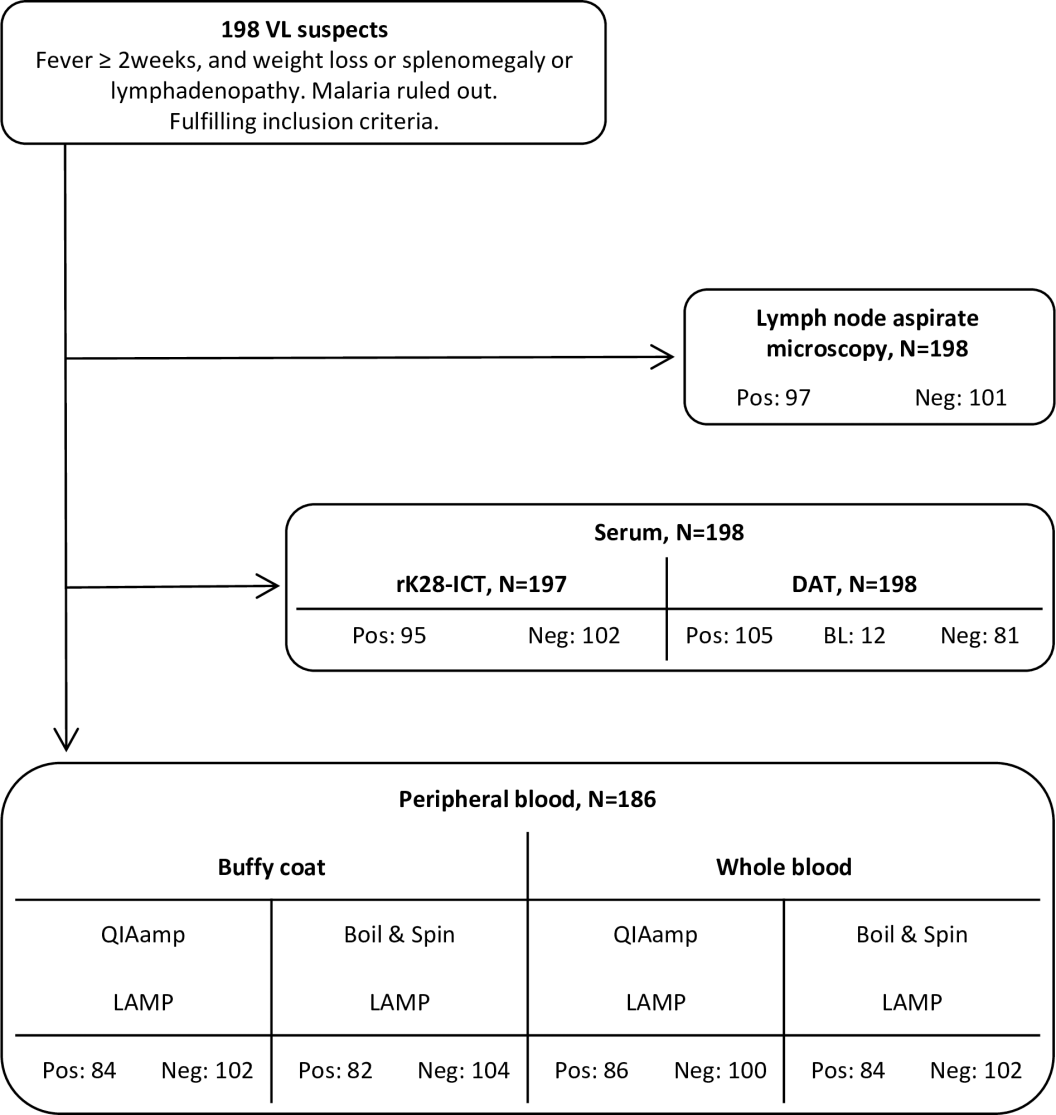
STARD diagram describing the flow of samples from VL suspects in the study.

A total of 198 VL suspects were included in the study. Fig 2 shows the workflow of testing samples from VL suspects. Only 185 VL suspects were tested by all methods, as during the process, 12 peripheral blood samples were lost for LAMP and one serum sample was lost for rK28 RDT testing.

Ninety seven patients suspected of VL tested positive by LNA-M, and were thus considered as true VL cases. All other VL suspects were considered as non-cases, and therefore controls for purposes of the analysis. Table 1 shows the demographic and clinical data from the 198 patients suspected of VL that were included in the study. Most of the cases were males younger than 15 years, with lymphadenopathy (89.7%), splenomegaly (89.7%), hepatomegaly (66.0%) and weight loss (54.6%) as the main clinical signs and symptoms. Table 2 shows the results of the different diagnostic tests in the group of 185 VL suspects that were tested by all methods. The results were quite similar to those obtained when data from all the 198 VL suspects were analysed together (S2 Table). No invalid result was recorded for the rK28 RDT, and the VL case missed by this test was found to be positive both by LAMP (any sample and sample preparation method) and DAT. Twelve patients had borderline results (BL) by DAT, eight of these were negative by microscopy, serology and LAMP, while the other four patients all had positive results in the tests applied. We conducted two separate analyses, in which we considered borderline results as either negative or positive.

**Table 1:** Clinical and demographic data of patients suspected of VL, distributed as case and controls after lymph node aspirate microscopy, participating in this study.

	Controls, n = 101 (%)	Cases, n = 97 (%)
Study site		
IEND	18 (17.8%)	24 (24.7%)
Bazoura	83 (82.2%)	73 (75.3%)
Female	48 (47.5%)	27 (27.8%)
Male	53 (52.5%)	70 (72.2%)
Age (years)		
< 5	4 (3.9)	11 (11.3)
5–15	34 (33.7)	43 (44.3)
16–40	52 (51.5)	37 (38.2)
41–60	9 (8.9)	5 (5.2)
>60	2 (2.0)	1 (1.0)
Fever > 2 weeks	101 (100)	97 (100)
Lymphadenopathy	67 (66.3)	87 (89.7)
Splenomegaly	35 (34.7)	87 (89.7)
Mean spleen size (cm)	3.8	8.2
Hepatomegaly	22 (21.8)	64 (66.0)
Mean liver size (cm)	3	3
Weight loss	45 (44.5)	53 (54.6)
Mean haemoglobin count (g/dl)	9.5	8.3
Jaundice	7 (6.9)	17 (17.5)
Cough	23 (22.8)	46 (47.4)

**Table 2:** Sensitivity, specificity, and positive and negative predictive values of the different diagnostic tests compared to lymph node aspirate microscopy (LNA-M), in a group of 185 VL suspects tested by all methods.

	Controls, n = 101 (LNA-M = Negative)		Cases, n = 84 (LNA-M = positive)		SE % [95% CI]	SP % [95% CI]	PPV % [95% CI]	NPV % [95% CI]
	Neg	Pos	Neg	Pos				
**rK28 RDT**	101	0	1	83	98.81 [95.89–100]	100 [99.50–100]	100 [99.40–100]	99.02 [96.62–100]
**DAT (BL = NEG)**	79	22	10	74	88.10 [80.57–95.62]]	78.22 [69.67–86.76]	77.08 [68.15–86.01]	88.76 [81.64–95.89]
**DAT (BL = POS)**	71	30	7	77	91.67 [85.16–98.17]	70.30 [60.89–79.70]	71.96 [62.98–80.94]	91.03 [84.04–98.01]
**LAMP-WB B&S**	100	1	2	82	97.62 [93.76–100]	99.01 [96.58–100]	98.80 [95.85–100]	98.04 [94.86–100]
**LAMP-WB QIA**	100	1	0	84	100 [99.40–100]	99.01 [96.58–100]	98.82 [95.94–100]	100 [99.50–100]
**LAMP-BC B&S**	100	1	4	80	95.24 [90.09–100]	99.01 [96.58–100]	98.77 [95.74–100]	96.15 [91.98–100]
**LAMP-BC QIA**	100	1	2	82	97.62 [93.76–100]	99.01 [96.58–100]	98.80 [95.85–100]	98.04 [94.86–100]

LNA-M: lymph node aspirate microscopy. SE: sensitivity. SP: specificity. PPV: positive predictive value. NPV: negative predictive value. CI: confidence interval. RDT: rapid diagnostic test. DAT (BL-NEG): DAT results considering borderline results as negative. DAT (BL-POS): DAT results considering borderline results as positive. LAMP-WB B&S: LAMP test using whole blood processed by the boil & spin method. LAMP-WB QIA: LAMP test using whole blood processed by the QIAgen kit. LAMP-BC B&S: LAMP test using buffy coat processed by the boil & spin method. LAMP-BC QIA: LAMP test using buffy coat processed by the QIAgen kit.

**Fig 3:**
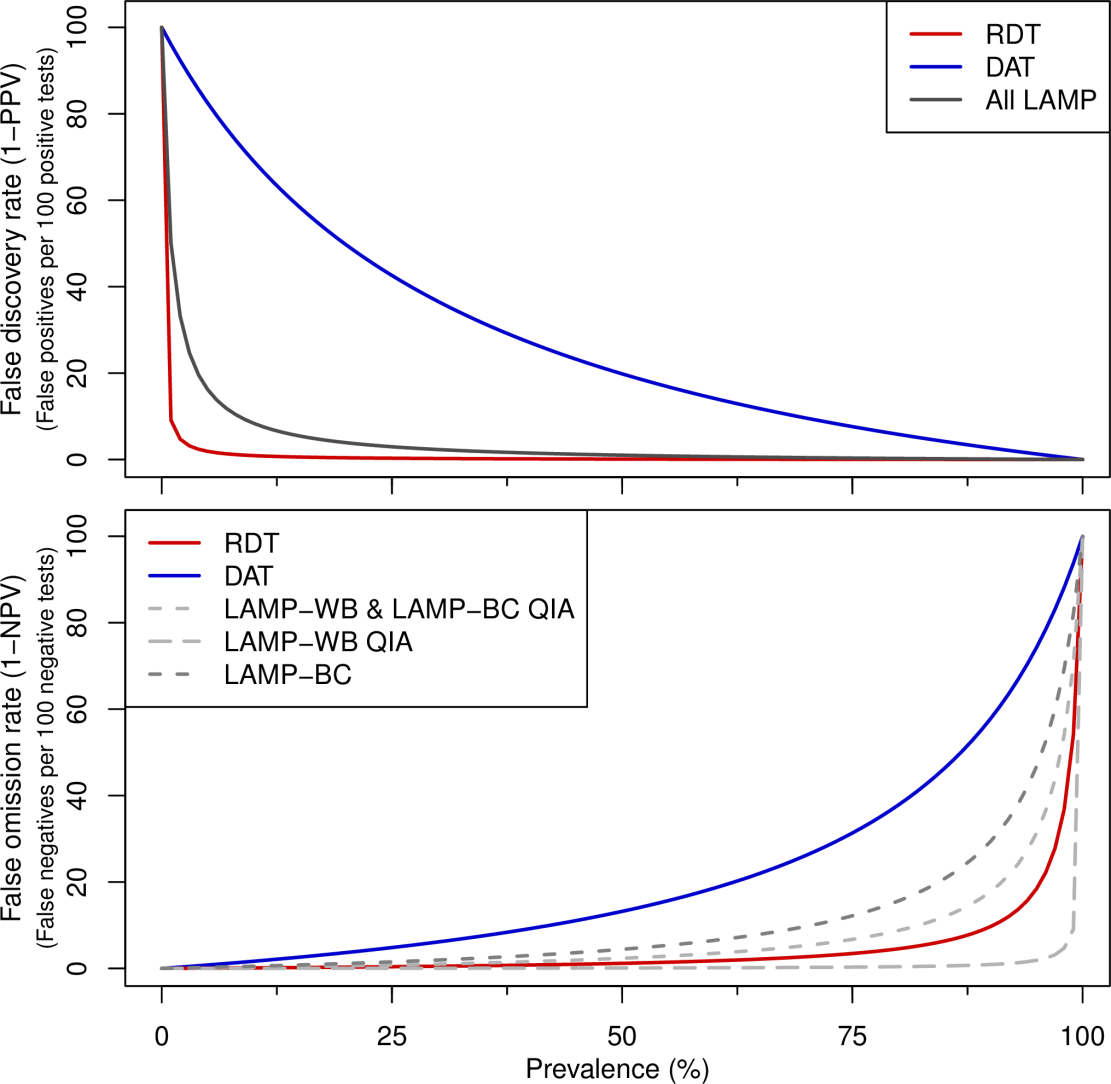
Plots of the false discovery rate and false omission rate with varying prevalence. Note that for RDT the sensitivity is set to an arbitrary 99.9% and for LAMP-WB QIA specificity set to 99.9% from 100%.

Of the two serological test used, the rK28 RDT presented higher sensitivity (98.81%) and specificity (100%) than DAT, considering borderline results as either negative (88.10% and 78.22%) or positive (91.67% and 70.30%). The Loopamp *Leishmania* Detection Kit had high specificity (99.01%), regardless of the type of sample tested (whole blood or buffy coat) or the sample processing method. The sensitivity was also high, with the best results obtained with whole blood processed either by the Boil & Spin method (97.62%) or by the QIAgen columns (100%). There were no significant differences between the sensitivities or specificities of LAMP in whole blood vs. buffy coat, being these processed either with the Boil & Spin method (WB and BC) or with the QIAamp DNA Mini Kit (WB QIA and BC QIA), McNemar's p > 0.1. At low prevalence, the performance of LAMP is similar to the RDT in terms of the numbers of false positives that are detected (Fig 3).

Any of the LAMP methods as well as the RDT had a very good agreement with LNA-M (*k* = 0.96–0.99), while the agreement between DAT and LNA-M was inferior (*k* = 0.60–0.65, good agreement). More details on the concordance of the different tests are provided in S3 Table).

## Discussion

Loop-mediated isothermal amplification (LAMP) is a nucleic acid amplification test that combines rapidity, simplicity, and high specificity. Since the test was first developed by Notomi *et al*. several LAMP tests have been developed to aid in the diagnosis of infectious and hereditary diseases [12,13]. The WHO has recently endorsed a LAMP test developed by Eiken Chemical Co. for use in the diagnosis of tuberculosis, and another test developed by this company has shown a high performance in the diagnosis of malaria [17–19].

Takagi *et al*. were the first to show the feasibility of diagnosing VL and post-kala azar dermal leishmaniasis (PKDL) using LAMP [20]. Since then, a number of other LAMP tests have been developed and applied to different uses, including screening of *Leishmania* infection in sandflies, diagnosis of canine leishmaniasis and detection of human asymptomatic infection [21–25]. Kothalawala *et al*. used a LAMP test targeting the kDNA minicircles for the diagnosis of cutaneous leishmaniasis (CL) in Sri Lanka, which had 82.6% sensitivity and 100% specificity when compared to microscopy [26]. A lower sensitivity (58.2%) was obtained by Nzelu *et al*. using a LAMP test targeting the *18S rRNA* gene for the diagnosis of CL and mucocutaneous leishmaniasis in Peru, but the specificity was not determined [27]. Other studies evaluating LAMP for VL diagnosis have shown a good diagnostic performance using different types of samples, of more than 90% sensitivity and up to 100% specificity (Table 3). To the best of our knowledge, the Loopamp *Leishmania* Detection Kit is the first LAMP test, available as a kit, which has been evaluated for VL diagnosis. It is in a ready-to-use format, with an additional advantage of being based on dried reagents, and therefore does not need a cold chain for transport and storage. The kit has a shelf life of one year if stored between 1 and 30°C, and stability testing has shown that it remains stable for 12 months at 30°C. It contains a primer combination targeting two different regions of the *Leishmania* genome, which makes this test *Leishmania* genus specific and can be used across the different endemic regions to diagnose all forms of leishmaniasis.

**Table 3:** Details and summary of the diagnostic performance of different studies using LAMP for VL diagnosis compared to our study.

	Studies				
	Ghasemian *et al*. [28]^a^	Verma *et al*. [29]^b^	Verma *et al*. [30]^c^	Khan *et al*. [31]^d^	This study^e^
**Country**	Iran	India	India	Bangladesh	Sudan
**LAMP target**	kDNA	kDNA	kDNA	kDNA	kDNA and *18SrRNA*
**Primer specificity**	*L*. *infantum*	*L*. *donovani*	*L*. *donovani*, *L*. *major*, *L*. *tropica*	*L*. *donovani*	*Leishmania* genus
**Study population**	47 VL cases 40 controls	55 VL cases 68 controls	66 VL cases 100 controls	75 VL cases 101 controls	185 VL suspects
**Clinical specimen tested**	Buffy coat	Whole blood BMA	Whole blood BMA	Buffy coat	Whole blood Buffy coat
**DNA purification method**	QIAamp Mini Kit (QIAgen)	QIAamp Mini Kit (QIAgen)	QIAamp Mini Kit (QIAgen)	QIAamp Mini Kit (QIAgen)	QIAamp Mini Kit (QIAgen) and Boil & Spin
**Reference test**	DAT (>1:3200) and BMA microscopy	BMA microscopy and qPCR on blood and BMA	rK39 RDT-positive patients confirmed by qPCR	Spleen aspirate microscopy	Lymph node aspirate microscopy
**Sensitivity**	BC 93.60%	WB 96.4% BMA 100%	WB 96.6% BMA 100%	BC 90.70%	WB-B&S 97.6% WB-QIA 100% BC-B&S 95.2% BC-QIA 97.6%
**Specificity**	100%	98.5%	100%	90.7%	99.01

^a^ Controls in [28]: 30 healthy controls from non-endemic area and 10 non-VL patients (malaria, TB, toxoplasmosis, hepatitis herpes virus). ^b^ Controls in [29]: 34 HC-E, 5 malaria, 5 TB, 18 leprosy, 6 fungal diseases. ^c^ Controls in [30]: 24 HC-E, 38 HC-NE, 7 malaria, 7 TB, 18 leprosy, 6 fungal diseases. ^d^ Controls in [31]: 25 HC-E, 26 HC-NE, 25 TB, 25 other diseases. ^e^ Data from the 185 VL suspects tested by all methods.

BC: buffy coat. BMA: bone marrow aspirate. B&S: boil & spin. DAT: direct agglutination test. HC-E: healthy controls from endemic region. HC-NE: healthy controls from non-endemic region. kDNA: kDNA minicircles. QIA: QIAamp DNA Mini Kit. VL: visceral leishmaniasis. WB: whole blood.

The rK28 RDT included in this study (OnSite *Leishmania* rK39-Plus, CTK Biotech, Inc., USA) presented a very good diagnostic performance, 98.81% sensitivity and 100% specificity. Similar results were obtained by Mukhtar *et al*. in a previous study in Sudan, where the rK28 RDT had 94.5% sensitivity and 97.6% specificity in serum samples and 92.5% and 100% when using whole blood [32].

**Fig 4:**
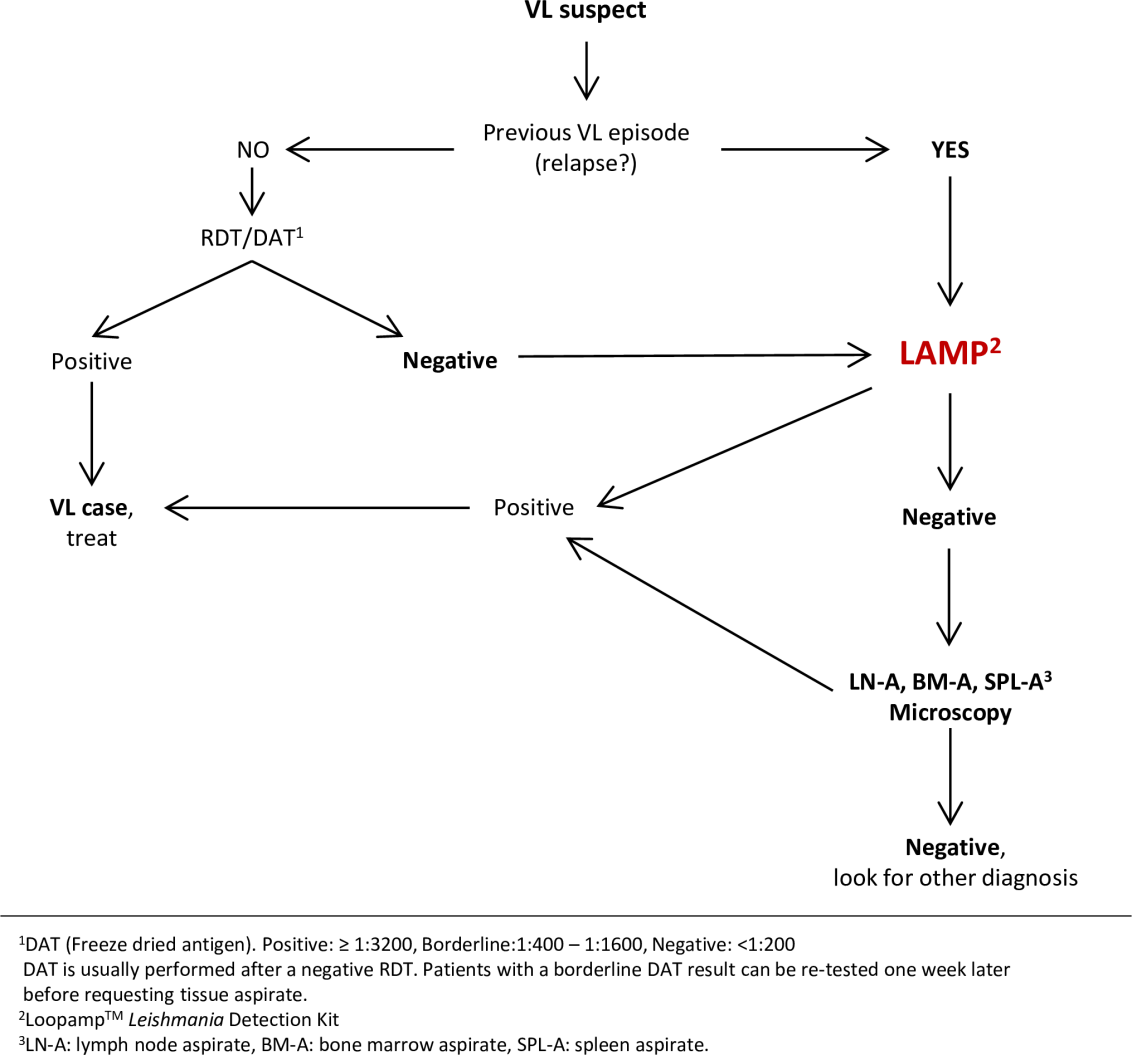
Role of Loopamp *Leishmania* Detection Kit in the VL diagnostic algorithm.

The excellent performance of rK28 RDT makes it ideal for primary diagnosis of VL. We have also shown that the performance of LAMP using peripheral blood is excellent. It is a rapid method that maintains simplicity throughout all steps, from extraction of DNA, to detection of amplification. A sensitivity and specificity of 97.6% and 99.1% can be obtained with the simple Boil & Spin method. All this indicates that Loopamp *Leishmania* Detection Kit can be included in the algorithm for diagnosis of VL in settings that are lower than the reference laboratory, replacing the need for invasive lymph node aspiration when parasite confirmation is needed in 97.6% of the cases using the Boil & Spin method for sample preparation (99.1% using QIAamp DNA Mini kit). Given the high performance of the rK28 RDT in this study LAMP would support diagnosis in cases where serology is profitless: VL suspects testing negative by serology, diagnosis of relapses or second VL episodes, and as a test of cure (Fig 4).

## Supporting information

S1 Table STARD checklist. (DOCX)

S2 Table Sensitivity, specificity, and positive and negative predictive values of the different diagnostic tests compared to lymph node aspirate microscopy (LNA-M), in the 198 VL suspects tested. (DOCX)

S3 Table Concordance (Cohen’s Kappa coefficient) between lymph node aspirate microscopy and the different tests in the group of 185 VL suspects examined by all diagnostic tests. (DOCX)
