# Supplementary material for: Sensitive and less invasive confirmatory diagnosis of visceral leishmaniasis in Sudan using loop-mediated isothermal amplification (LAMP)
Source: PLoS Negl Trop Dis. 2018 Feb 14;12(2):e0006264. doi: 10.1371/journal.pntd.0006264 (PMC5828521; doi:10.1371/journal.pntd.0006264)
Supplement: S2 Table — (DOCX) [file pntd.0006264.s002.docx]

Table S2. *Sensitivity, specificity, and positive and negative predictive values of the different diagnostic tests compared to lymph node aspirate microscopy (LNA-M), in the 198 VL suspects tested.*

| Table S2. *Sensitivity, specificity, and positive and negative predictive values of the different diagnostic tests compared to lymph node aspirate microscopy (LNA-M), in the 198 VL suspects tested.* | | | | | | | | |
| --- | --- | --- | --- | --- | --- | --- | --- | --- |
|  | **Controls, n=101**  **(LNA-M= Negative)** | | **Cases, n=97**  **(LNA-M= positive)** | | **SE % [95% CI]** | **SP % [95% CI]** | **PPV % [95% CI]** | **NPV % [95% CI]** |
|  | **Neg** | **Pos** | **Neg** | **Pos** |  |  |  |  |
| **rK28-RDT (n=197)** | 101 | 0 | 1 | 95 | 98.96 [96.41-100.00] | 100.00 [99.50-100.00] | 100.00 [99.47-100.00] | 99.02 [96.62-100.00] |
| **DAT (BL=NEG) (N=198)** | 79 | 22 | 14 | 83 | 85.57 [78.06-93.08] | 78.22 [69.67-86.76] | 79.05 [70.79-87.31] | 84.95 [77.14-92.75] |
| **DAT (BL=POS) (N=198)** | 71 | 30 | 10 | 87 | 89.69 [83.12-96.26] | 70.30 [60.89-79.70] | 74.36 [66.02-82.70] | 87.65 [79.87-95.44] |
| **LAMP-WB B&S (N=186)** | 100 | 1 | 2 | 83 | 97.65 [93.84-100.00] | 99.01 [96.58-100.00] | 98.81 [95.89-100.00] | 98.04 [94.86-100.00] |
| **LAMP-WB QIA (N=186)** | 100 | 1 | 0 | 85 | 100.00 [99.41-100.00] | 99.01 [96.58-100.00] | 98.84 [95.99-100.00] | 100.00 [99.50-100.00] |
| **LAMP-BC B&S (N=186)** | 100 | 1 | 4 | 81 | 95.29 [90.20-100.00] | 99.01 [96.58-100.00] | 98.78 [95.80-100.00] | 96.15 [91.98-100.00] |
| **LAMP-BC QIA (N=186)** | 100 | 1 | 2 | 83 | 97.65 [93.84-100.00] | 99.01 [96.58-100.00] | 98.81 [95.89-100.00] | 98.04 [94.86-100.00] |

*LNA-M: lymph node aspirate microscopy. SE: sensitivity. SP: specificity. PPV: positive predictive value. NPV: negative predictive value. CI: confidence interval. RDT: rapid diagnostic test. DAT (BL-NEG): DAT results considering borderline results as negative. DAT (BL-POS): DAT results considering borderline results as positive. LAMP-WB B&S: LAMP test using whole blood processed by the boil & spin method. LAMP-WB QIA: LAMP test using whole blood processed by the QIAgen kit. LAMP-BC B&S: LAMP test using buffy coat processed by the boil & spin method. LAMP-BC QIA: LAMP test using buffy coat processed by the QIAgen kit.*
