# Supplementary material for: Sensitive and less invasive confirmatory diagnosis of visceral leishmaniasis in Sudan using loop-mediated isothermal amplification (LAMP)
Source: PLoS Negl Trop Dis. 2018 Feb 14;12(2):e0006264. doi: 10.1371/journal.pntd.0006264 (PMC5828521; doi:10.1371/journal.pntd.0006264)
Supplement: S3 Table — (DOCX) [file pntd.0006264.s003.docx]

Table S3. *Concordance (Cohen’s Kappa coefficient) between lymph node aspirate microscopy and the different tests in the group of 185 VL suspects examined by all diagnostic tests.*

| Table S3. Concordance (Cohen’s Kappa) between lymph node aspirate microscopy and the different in the group of 185 VL suspects examined by all diagnostic tests. | | | | | | |
| --- | --- | --- | --- | --- | --- | --- |
|  | **Agreement**  **Observed/Expected** | **kappa** | **kappa 95% CI** | **Minimum kappa** | **Maximum kappa** | **p-value** |
| **rK28-RDT** | 0.9946/0.5047 | 0.9891 | 0.9678-1.0000 | -0.0027 | 0.9892 | 0.0000 |
| **DAT (BL=NEG)** | 0.8270/0.4983 | 0.6553 | 0.5475-0.7630 | -0.0947 | 0.6641 | 0.0000 |
| **DAT (BL=POS)** | 0.8000/0.4928 | 0.6057 | 0.4954-0.7160 | -0.1111 | 0.6154 | 0.0000 |
| **LAMP-WB B&S** | 0.9838/0.5047 | 0.9673 | 0.9305-1.0000 | -0.0082 | 0.9676 | 0.0000 |
| **LAMP-WB QIA** | 0.9946/0.5037 | 0.9891 | 0.9678-1.0000 | -0.0027 | 0.9892 | 0.0000 |
| **LAMP-BC B&S** | 0.9730/0.5057 | 0.9453 | 0.8981-0.9926 | -0.0137 | 0.9460 | 0.0000 |
| **LAMP-BC QIA** | 0.9838/0.5047 | 0.9673 | 0.9305-1.0000 | -0.0082 | 0.9676 | 0.0000 |

*CI: confidence interval. RDT: rapid diagnostic test. DAT (BL-NEG): DAT results considering borderline results as negative. DAT (BL-POS): DAT results considering borderline results as positive. LAMP-WB B&S: LAMP test using whole blood processed by the boil & spin method. LAMP-WB QIA: LAMP test using whole blood processed by the QIAgen kit. LAMP-BC B&S: LAMP test using buffy coat processed by the boil & spin method. LAMP-BC QIA: LAMP test using buffy coat processed by the QIAgen kit.*
